# Are Fusion Transcripts in Relapsed/Metastatic Head and Neck Cancer Patients Predictive of Response to Anti-EGFR Therapies?

**DOI:** 10.1155/2017/6870614

**Published:** 2017-11-12

**Authors:** Paolo Bossi, Marco Siano, Cristiana Bergamini, Maria Cossu Rocca, Andrea P. Sponghini, Marco Giannoccaro, Luca Tonella, Alessandro Paoli, Edoardo Marchesi, Federica Perrone, Silvana Pilotti, Laura D. Locati, Silvana Canevari, Lisa Licitra, Loris De Cecco

**Affiliations:** ^1^Head and Neck Medical Oncology Unit, Fondazione IRCCS Istituto Nazionale dei Tumori, Milan, Italy; ^2^Department of Internal Medicine, Clinic for Medical Oncology, Cantonal Hospital St. Gallen, St. Gallen, Switzerland; ^3^Division of Medical Oncology, European Institute of Oncology, Milan, Italy; ^4^SC of Oncology, AOU Maggiore della Carità, Novara, Italy; ^5^Functional Genomics and Bioinformatics, Department of Applied Research and Technology Development, Fondazione IRCCS Istituto Nazionale dei Tumori, Milan, Italy; ^6^Laboratory of Experimental Molecular Pathology, Department of Diagnostic Pathology and Laboratory, Fondazione IRCCS Istituto Nazionale dei Tumori, Milan, Italy; ^7^University of Milan, Milan, Italy

## Abstract

Prediction of benefit from combined chemotherapy and the antiepidermal growth factor receptor cetuximab is a not yet solved question in head and neck squamous cell carcinoma (HNSCC). In a selected series of 14 long progression-free survival (PFS) and 26 short PFS patients by whole gene and microRNA expression analysis, we developed a model potentially predictive of cetuximab sensitivity. To better decipher the “omics” profile of our patients, we detected transcript fusions by RNA-seq through a Pan-Cancer panel targeting 1385 cancer genes. Twenty-seven different fusion transcripts, involving mRNA and long noncoding RNA (lncRNA), were identified. The majority of fusions (81%) were intrachromosomal, and 24 patients (60%) harbor at least one of them. The presence/absence of fusions and the presence of more than one fusion were not related to outcome, while the lncRNA-containing fusions resulted enriched in long PFS patients (*P* = 0.0027). The CD274-PDCD1LG2 fusion was present in 7/14 short PFS patients harboring fusions and was absent in long PFS patients (*P* = 0.0188). Among the short PFS patients, those harboring this fusion had the worst outcome (*P* = 0.0172) and increased K-RAS activation (*P* = 0.00147). The associations between HNSCC patient's outcome following cetuximab treatment and lncRNA-containing fusions or the CD274-PDCD1LG2 fusion deserve validation in prospective clinical trials.

## 1. Introduction

The therapeutic opportunities for recurrent and/or metastatic (RM) head and neck squamous cell carcinoma (HNSCC) may be divided into 3 modalities: (i) potentially salvageable treatments, like (re)irradiation or salvage surgery; (ii) palliative systemic therapies, such as chemotherapy and/or targeted agents; and (iii) the best supportive care. Salvage therapies are the first options, but their feasibility is limited by the patient's performance status or by other prognostic factors as disease-free interval as well as by technical aspects such as the site and extension of disease or to the previous administered treatments [1–3].

First line palliative systemic therapy is represented by the combination of platinum-based chemotherapy and cetuximab, an antiepidermal growth factor receptor (EGFR) agent. This combination, as shown in the pivotal EXTREME trial, is able to achieve a clinical response in more than one-third of the patient, and it is able to statistically improve overall survival (OS) and progression-free survival (PFS) and it improves the patient's quality of life, when compared to chemotherapy alone [4]. However, the median OS is of 10.1 months with more than 80% of the patients experiencing one grade 3 or 4 adverse event. Thus, the presence of multiple mechanisms of intrinsic resistance to this therapeutic combination exposes some patients to the double-negative effect of drug toxicity and disease unresponsiveness.

Therefore, the issue of predicting which patient will benefit from this approach is an outstanding question in head and neck oncology. In fact, all the efforts in identifying specific alteration in EGFR status (studied by immunohistochemistry, amplification, or mutation) did not reach their purpose [5–7].

A solution might, however, lie with a molecular approach [8, 9], judging also from the encouraging results of our predictive models, developed to test cetuximab sensitivity in RM-HNSCC patients [10, 11].

Specifically, we implemented a model of cetuximab and chemotherapy (CT) sensitivity analyzing the 2 extremities of responsiveness to the drugs, represented by patients achieving a long PFS, defined to be more than 1 year, and patients showing a short PFS, defined to be less than 5.6 months, that is, the median PFS of the EXTREME trial. Using these selected patient cohorts and applying, as first, gene expression analysis [10] and in a second study an integrative analysis of miRNA and mRNA expression [11], we identified specific profiles corresponding to the long and short PFS [10] and a height miRNA gene-integrated signature with an excellent accuracy in predicting treatment response [11]. Trying to better decipher the different biological molecular characteristics of the extremities of the response curve to cetuximab CT, we decided to explore the new area of fusion transcripts in the search of other complementing genomics.

Gene fusions could occur by structural rearrangements or by transcription read-through of neighboring genes, being the second mechanism responsible for a large proportion of gene fusions (see [12] for a recent computationally oriented literature review); their clinical utility in cancer as biomarkers for prognosis or diagnosis is proven, and some fusion proteins are promising therapeutic targets (see [13] for the landscape of cancer-associated transcript fusions). At present, 300 samples of the TCGA-HNSCC dataset were characterized for the presence of transcript fusions [12], and among the identified fusion events, FGFR3-TACC3 fusion was detected in two HPV-positive tumors. Subsequently, in a HNSCC cell model system [14], the signaling by FGFR3-TACC3 fusion protein was further characterized as a novel mechanism of resistance to EGFR/ERBB3 inhibition. A limited number of other reports focused on gene fusions in HNSCC samples [15] or cell lines [16–17].

No data are presently available in a clinical setting about a possible association between gene fusion presence and response to a targeted therapy, such as the one with EGFR inhibitors. Taking again the advantage of the RM-HNSCC clinical material already genomically characterized by us [10, 11], we looked for the expression of fusion transcripts derived from 1385 different genes, selected on the basis of their putative role in cancer, as potential markers of intrinsic sensitivity/resistance to cetuximab CT.

## 2. Materials and Methods

### 2.1. Patients and Study Design

Forty formalin-fixed paraffin-embedded (FFPE) tumor specimens from RM-HNSCC patients treated between 2008 and 2012 with first-line platinum and cetuximab-based combination were collected and divided according to PFS following cetuximab CT treatment in long (14 patients) and short PFS (26 patients) as detailed in [10]. Briefly, the two groups were balanced for known prognostic factors [18] (primary tumor site, performance status, weight loss, prior radiotherapy, tumor grade, residual disease at primary tumor site, age, and gender). Long PFS had a median PFS of 19 months (range 12–36) while short PFS had a median PFS of 3 months (range 1–5.5).

### 2.2. Transcript Fusion Detection

To detect transcript fusions in RM-HNSCC, the TruSight RNA Pan-Cancer panel (Illumina) targeting 1385 cancer genes, including 507 known genes involved in fusions and 878 genes either mutated or deregulated in cancers, was used according to the provider's protocol. The panel design covers all exons and 160 bp at the 5′ and 3′ UTR of every gene. Briefly, cDNA is generated from 50 ng of total RNA from the FFPE specimens using random priming. After second strand synthesis, sequencing adapters are ligated to the double-stranded cDNA fragments. The coding regions of expressed cancer-associated genes were captured from 200 ng of this library using sequence-specific probes to create the final sequencing library. Quality check was performed using 4200 TapeStation and D1000 ScreenTape Assays (Agilent) yielding libraries with a band peak at ~250–300 bp. Samples were equimolarly pooled and sequenced on a NextSeq500 sequencer using the NextSeq500 High Output Kit v2 (150 cycles) chemistry (Illumina) to obtain 40 M/sample paired end reads of length 2 × 75 bps.

The data processing was performed on BaseSpace Sequence Hub, a dedicated genomics computing environment for data management and analysis applying TopHat Alignment v1.0. TopHat Alignment workflow allows the following functions: (i) read mapping on homo sapiens UCSC hg19 through the TopHat 2 aligner and (ii) fusion calling with TopHat-Fusion [19]. After the alignment of sequencing reads within the exon regions, the reads not entirely aligned were divided into multiple segments of 25 bp. It is expected that the initially unmapped reads contain sequencing portions residing on different chromosomes or on the same chromosome but, after rearrangement, representing potential fusion candidates. The first and last 25 bp portions were aligned on the genome through Bowtie. When an alignment pattern is detected, the entire read sequence is used to identify the fusion point by stitching segments to obtain the full read alignment. The oligo capture approach of the TruSight RNA Pan-Cancer panel allows pulling down one target gene among the 1385 genes in the panel and the partner fusion not necessarily included in the panel. Since the TopHat-fusion algorithm works independently of the information about known genes, it can also lead to the identification of novel fusion products. To avoid false positive calls, candidate gene fusions were filtered out imposing the following parameters: (i) intrachromosome fusions have to be separated by 100.000 bp distance; (ii) spanning reads on both sides should have at least 13 bp; and (iii) reads map to multiple locations (>2). The annotated gene fusions were then displayed using the OmicCircos software package [20] with respect to genomic position using the hg19 reference.

### 2.3. Characteristics of Genes Present in Transcript Fusion

The information on genes/lncRNA was retrieved from https://www.ncbi.nlm.nih.gov/gene and https://lncipedia.org/ [21] (version 4.1, May 4, 2017, containing 146,742 human-annotated lncRNAs) and http://cancer.sanger.ac.uk/cosmic and https://cancergenome.nih.gov/.

The presence of fusion transcripts in cancers was searched in the following websites: Pubmed (https://www.ncbi.nlm.nih.gov/pubmed) and TCGA fusion gene data portal (http://54.84.12.177/PanCanFusV2/).

### 2.4. Functional Analysis

To disclose the molecular pathways associated with CD274/PDCD1LG2 fusion, we retrieved gene expression data from Bossi et al. [10] deposited on GEO repository (GSE65021). Gene set enrichment was investigated by gene set enrichment analysis (GSEA) [22] analyzing seven oncogenic signatures found in our previous studies [10, 11] and including *β*-catenin, E2F3, EGFR, KRAS, MYC, NOTCH, and p53. To graphically represent the significant gene sets and to display their enrichment significance, we used Enriched Map implemented as a Java plugin for the freely available Cytoscape network visualization and analysis software [23].

### 2.5. Statistical Analysis

The presence of fusions in long and short PFS as well as of specific fusions was evaluated using the Fisher exact test through GraphPad Prism software package. A *P* value equal or <0.05 was considered to indicate statistical significance. Differences in PFS between patients harboring or not CD274/PDCD1LG2 fusion among the 26 short PFS patients were assessed using log-rank test and R package survival [24].

## 3. Results and Discussion

With the aim to disclose the biological features associated with cetuximab sensitivity in RM-HNSCC, we applied an RNA-seq approach through a Pan-Cancer panel to a selected cohort [40 patients treated with platinum- and cetuximab-based combination and having long PFS (*n* = 14) and short PFS (*n* = 26)].

**Figure 1:**
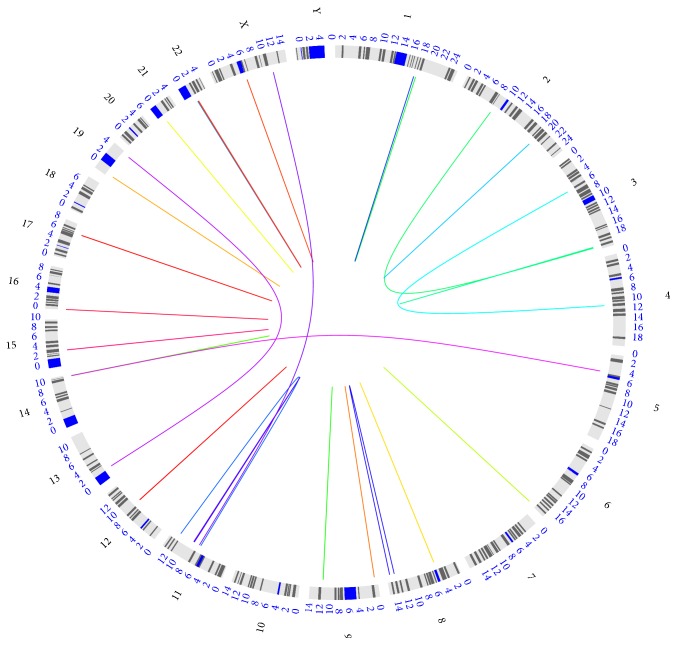
Circos plot of the genomic landscape of gene fusions identified by RNA-seq in our 40 RM-HNSCC samples. The outer ring displays the chromosome ideograms. The fusion transcripts are shown as line arcs linking the two genomic loci.

Based on the applied workflow of analysis, 27 different fusion transcripts were identified; Figure 1 shows the genomic landscape of the identified transcript fusions that is further detailed in Supplementary Table 1 available online at https://doi.org/10.1155/2017/6870614. Twenty-two out of twenty-seven (81%) fusion transcripts were intrachromosomal and located in neighboring genes while 5 resulted from structural rearrangements and translocations to a different chromosome. The identified transcript fusions involved rearrangements in all, but Chr7, Chr10, Chr18, Chr20, and ChrY, chromosomes; Chr3, Chr11, and Chr22 harbor three different fusions and high level of gains (ratio > 1.5) at 11q13, and their association with poor survival has been described in HNSCC (see [25]); Chr1, Chr2, Chr8, Chr9, Chr14, and Chr19 harbor two fusions. The total number and chromosomal distribution of the identified fusion transcripts are essentially in agreement with data already reported in HNSCC samples [13–15] even if the comparison is difficult due to the different approach adopted (targeted versus whole genome) (see below). In all the HNSCC studies on clinical samples, including the present one, fusion transcripts resulting from translocations are relatively rare while the majority is generated by nonstructural rearrangement mechanisms, such as transcription read-through of neighboring genes or splicing of mRNA molecules. This type of gene fusions is reported to be preferentially derived by genomic instability (see [12] for review of the mechanisms).

Twenty-four patients (60%) harbor at least one of the 27 identified transcript fusions; the presence and main characteristics of gene fusions detected in each RM-HNSCC patient of our selected case material are reported in Table 1 (see [10] for clinical pathologic characteristics of the patients). We investigated whether the presence of transcript fusions is associated with long or short PFS under cetuximab treatment: 10/14 (71%) long PFS cases and 14/26 (54%) short PFS harbor at least one fusion; however, the presence or absence of fusions is not significantly related with outcome (Table 2). The chromosomal rearrangements in cancer cells could also lead to multiple fusion events. Fifteen and 9 cases harbor only one transcript fusion and more than one, respectively (Table 2); in detail, 3 patients harbor 2 fusions, 4 patients 3 fusions, 1 patient 4 fusions, and 1 patient 6 fusions; nine fusions are present in two or more patients (Table 1). Since the coexistence of multiple fusions might mirror the extent of aberrations present in the tumor, we investigated whether the presence of more than one fusion is associated with outcome under cetuximab. Five out of the 10 long PFS and 4/14 short PFS cases presented more than one fusion, but this difference did not reach a significant level (Table 2). The accumulation of transcript fusions may be associated with tumor progression; since in our case material the RNA was obtained in 9 cases from samples taken at recurrence/metastasis, we analyzed whether in this subgroup of patients (9/40) the presence/number of transcript fusions was higher. Eight out of 9 recurrent/metastatic cases (89%) harbor at least one fusion compared to 16/31 (52%) cases from primary lesions; although the difference did not reach a significant level (*P* = 0.06), a trend was clearly appreciable.

The 27 transcript fusions involve both mRNA and lncRNA, being 21 mRNA-mRNA, 3 lncRNA-mRNA, 2 mRNA-lncRNA, and 1 lncRNA-lncRNA. The lncRNA-containing fusions are enriched in long PFS patients with 8/10 and 2/14 in long and short PFS cases, respectively, harboring a lncRNA fusion (*P* = 0.0027). Two fusions involving lncRNA, ENSG00000231669-MSN, and ENSG00000231121-NAV3, were each detected in three long PFS patients. At present, while lncRNAs are relatively well-characterized, being involved in the regulation of numerous cellular processes and being associated with cancer development and progression [26], little is known about their role in lncRNA-containing fusions. The current fusion-detection algorithms and bioinformatics pipelines are focused on recognizing fusion candidates mapping to protein-coding mRNA systematically omitting lncRNA [12]. As a result, only a handful of gene fusions containing lncRNAs has been reported [27]. Some studies highlighted a biological, functional, and even clinical relevance of specific mRNA/lncRNA fusions proving that these lncRNAs might contribute to the aberrant regulation of their partner [12]. The identification of lncRNA-containing fusions was achieved in our case material due to the adopted targeted approach. We selected this approach, instead of RNA-seq used with the 300 HNSCC [13] and the 47 oral squamous cell carcinoma (OSCC) [15], due to the availability of only archival FFPE samples whose RNA-seq analysis may result limited, as recently highlighted in another cancer type by direct comparison of paired frozen and FFPE samples [28].

Several studies of gene fusion networks have found that the majority of fusion genes partner with a single other gene, but it is known that some genes might recombine with multiple partners being the MLL the extreme example, described to fuse with over 60 different partner genes [29]. In our study, MLL was present in a single fusion while IGLL5 recombined with BMS1P20 and IGL1-40 (see Supplementary Table 1).

The distribution and the gene partners of our 27 fusion transcripts were compared with the 382 and the 282 fusions detected by a whole genome approach in HNSCC [13] and OSCC [15], respectively; we recorded a 33% of fusions shared among more patients and no overlap with fusions previously identified in HNSCC/OSCC. The different results could be mainly attributed to the use of a panel that enabled a higher sequencing depth but that was biased toward cancer genes. Despite these differences, one or both gene partners of our 27 fusions was/were present in association with other genes in the 7887 high confidence fusion transcripts identified in 4366 primary tumor samples from 13 tumor types including HNSCC (Supplementary Table 1).

We analyzed the characteristics of the gene partners (see details in Supplementary Table 1) to potentially define the molecular functions of the identified fusions, and we observed that chromatin modifiers (KMTA2-MLL, RCOR1, and KAT6A), kinases (RPS6KA, MUSK, TRIM28, MPZL1, and MAP2K2), and phosphates (LPAR1, PICALM, RPS6KB1, DLG2, PPP6RB, TPTA, and PI4KA) were frequently present.

**Figure 2:**
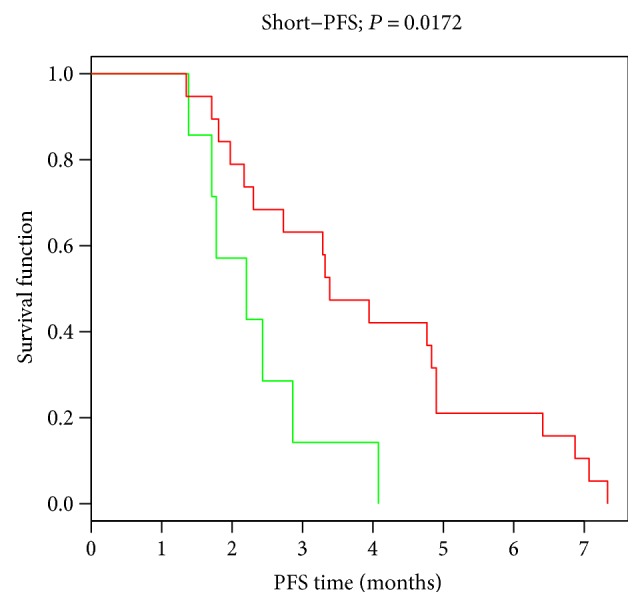
Kaplan-Meier curves showing PFS among patients with the presence or absence of the CD274-PDCD1LG2 fusion transcript. Median PFS: 2.2 months in the group with fusion (*n* = 7) and 3.4 months in the group not harboring the fusion (*n* = 19) (*P* = 0.0172).

**Figure 3:**
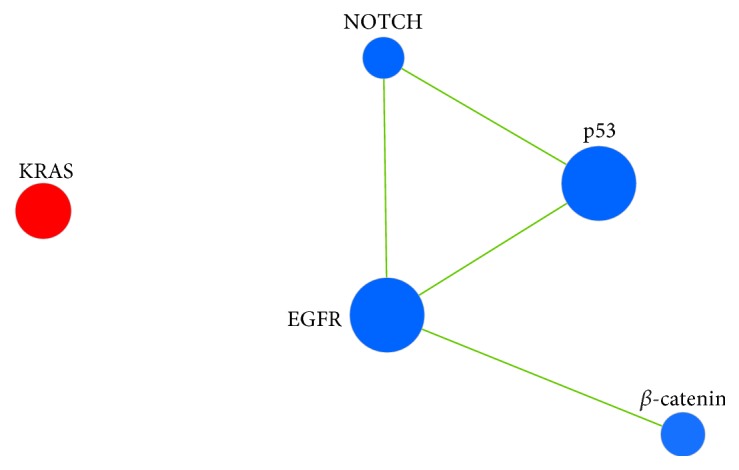
Enrichment map visualizing results of GSEA analysis for cases with the presence/absence of the CD274-PDCD1LG2 fusion. Seven oncogenic signatures inferred from our previous studies [10, 11] were tested, and five resulted a significant difference (oncogenic signature = node). Node size: number of genes in the gene set. Node color: red = enriched in cases harboring the CD274-PDCD1LG2 fusion. Blue = enriched in cases not harboring the fusion. Edges: connect significantly overlapping gene sets (width reflects the degree of the overlap).

Worth mentioning, the fusion CD274-PDCD1LG2 was present as single fusion (5/14) or associated with other fusions (2/14) in short PFS patients while it was absent in all long PFS patients (*P* = 0.0188). CD274-PDCD1LG2 fusion defined a subgroup into short PFS; in fact, by Kaplan-Meier analysis, the median estimates of PFS in patients harboring or not the fusion were 2.2 and 3.4 months, respectively (*P* = 0.0172 by the log-rank test) (Figure 2). Thus, we investigated the biology behind the CD274-PDCD1LG2 fusion in the short PFS cases analyzing molecular pathways through GSEA. The oncogenic signatures reported in our previous studies [10, 11] were tested for their enrichment in cases harboring CD274-PDCD1LG2 fusion, and as reported in the enrichment map (Figure 3), KRAS (*P* = 0.00147; NES = 1.62) is enriched in cases with the fusion, while EGFR (*P* = 10E‐04; NES = −2.04), p53 (*P* = 10E‐04; NES = −1.83), NOTCH (*P* = 10E‐04; NES = −1.82), and *β*-catenin (*P* = 10E‐04; NES = −2.04) onco-signatures are enriched in cases without the fusion. E2F3 and MYC are not significantly different between cases with the presence or absence of fusion.

Both partner genes are in the 9p24.1 locus, a region of recurrent structural and copy number alterations in hematologic tumors, and this fusion was present in 20% of primary mediastinal large B-cell lymphoma and in lower percent in other lymphomas [30]. Furthermore, in lymphomas, the rearrangement was significantly correlated with overexpression of PDL transcripts [30]. The products of the gene partners of our most frequent fusion transcript, CD274 and PDCD1LG2, also known as programmed death ligand-1 and 2 (PD-L1 and PD-L2), respectively, have been implicated in promoting tumor cell immune evasion acting as negative regulators of antitumor immunity by binding their cognate receptor, PD-1, on cytotoxic T-cells. No data are presently available about this fusion in other types of solid tumors, and our observation that RM-HNSCC cases harboring CD274-PDCD1LG2 fusion have poor prognosis and resistance to cetuximab deserve further analysis and validation in wider series of patients entered/entering in anti-EGFR-targeted trials.

Recently, high PD-L1 expression was identified as a strong prognostic factor of HNSCC patient's worse outcome [31]. Within clinical trials with immune checkpoint inhibitors (CPIs) in RM-HNSCC, higher response rates were noted in patients with higher PD-L1 expression [32, 33]. However, other PD-1 ligands could be crucial in determining the efficacy of CPIs. As it has been recently showed, the coexpression of both PD-L1 and PD-L2 in tumoral specimens of patients treated with pembrolizumab correlated with higher responsiveness to this drug [34]. Further investigation is required into the role of the CD274-PDCD1LG2 fusion as pharmacogenomics biomarker not only as a prognosticator in RM-HNSCC patients but also as a possible predictive biomarker of immunotherapy response to better select patients for a tailored treatment approach.

## 4. Conclusions

Transcript fusions resulting from chromosomal rearrangements are genetic alterations well-known from decades and they can in oncology (i) serve as diagnostic markers, (ii) provide insight into tumor biology, and (iii) serve as specific therapeutic targets. By an RNA-seq approach through a dedicated Pan-Cancer panel, we investigated the presence and role of transcript fusions as potential pharmacogenomic markers of RM-HNSCC patients' response to cetuximab and platinum-based chemotherapy. We identified 27 different fusion transcripts and observed significant associations between lncRNA-containing fusions and patient's better outcome and the presence of the CD274-PDCD1LG2 fusion and worst outcome. These observations deserve the testing in clinical trials but if confirmed, as seen with other gene fusions in other tumor entities, they could open the way to a more tailored therapeutic approach. Further on, combination treatments with only immune therapeutic approaches or with targeted agents or classic chemotherapy could be of major importance to increase efficacy and outcome in RM-HNSCC patients. In this regard, our findings are important to be acknowledged and could lead to further researches and new trial designs.

## Supplementary Material

Supplementary Table 1. Details of the 27 different fusion transcripts that identified based on the applied workflow of analysis.

## Figures and Tables

**Table 1:** Presence and main characteristics of gene fusions detected in each RM-HNSCC patient of our selected case material; see [10] for clinical pathologic characteristics of the patients.

Sample ID	Gene fusion	“Left” partner		“Right” partner	
		Gene	Chromosome	Gene	Chromosome
*Short PFS under chemotherapy-cetuximab treatment*					
GU05	No				
GU09	No				
GU10	Yes	DLG2	Chr11	PICALM	Chr11
		NUMA1	Chr11	GRIA3	ChrX
		ZMYM2	Chr13	TRIM28	Chr19
GU11	No				
GU13	Yes	CLTC	Chr17	RPS6KB1	Chr17
GU14	Yes	CD274	Chr9	PDCD1LG2	Chr9
GU15	No				
GU17	No				
GU18	No				
GU20	Yes	CD274	Chr9	PDCD1LG2	Chr9
GU21	No				
GU22	No				
GU23	No				
GU24	Yes	BMS1P20	Chr22	IGLL5	Chr22
GU25	Yes	CD274	Chr9	PDCD1LG2	Chr9
GU26	Yes	FGF12	Chr3	MB21D2	Chr3
GU27	No				
GU28	Yes	METTL13	Chr1	DNM3	Chr1
		CTNNA2	Chr2	HES1	Chr3
		RPS6KA2	Chr6	RNASET2	Chr6
		MUSK	Chr9	LPAR1	Chr9
		CD274	Chr9	PDCD1LG2	Chr9
		TRAF3	Chr14	ENSG00000259717	Chr14
GU29	Yes	CD274	Chr9	PDCD1LG2	Chr9
GU30	Yes	RCSD1	Chr1	MPZL1	Chr1
GU31	Yes	CD274	Chr9	PDCD1LG2	Chr9
		PPP6R3	Chr11	MLL	Chr11
GU34	Yes	NUMA1	Chr11	GRIA3	ChrX
		ZMYM2	Chr13	TRIM28	Chr19
GU38	No				
GU40	No				
GU41	Yes	PVT1	Chr8	ENSG00000253288	Chr8
GU43	Yes	CD274	Chr9	PDCD1LG2	Chr9
*Long PFS under chemotherapy cetuximab treatment*					
GU04	Yes	FLNB	Chr3	ENSG00000245384	Chr4
GU06	No				
GU07	Yes	METTL13	Chr1	DNM3	Chr1
		MUSK	Chr9	LPAR1	Chr9
		ENSG00000231669	ChrX	MSN	ChrX
GU08	Yes	ENSG00000231669	ChrX	MSN	ChrX
GU12	Yes	C9	Chr5	RCOR1	Chr14
		ENSG00000259446	Chr15	RYR3	Chr15
		IGLV1-40	Chr22	IGLL5	Chr22
GU16	Yes	WDR90	Chr16	RHOT2	Chr16
GU19	Yes	ANK1	Chr8	KAT6A	Chr8
		ZBTB7A	Chr19	MAP2K2	Chr19
GU32	Yes	ZBTB7A	Chr19	MAP2K2	Chr19
		TPTE	Chr21	BAGE2	Chr21
		ENSG00000231669	ChrX	MSN	ChrX
GU33	Yes	ENSG00000231121	Chr12	NAV3	Chr12
GU35	No				
GU36	Yes	ZC3H15	Chr2	ITGAV	Chr2
		PPP6R3	Chr11	MLL	Chr11
		ENSG00000231121	Chr12	NAV3	Chr12
		PI4KA	Chr22	CRKL	Chr22
GU37	Yes	ENSG00000231121	Chr12	NAV3	Chr12
GU39	No				
GU42	No				

**Table 2:** Summary of the gene fusions detected in patients treated with cetuximab and chemotherapy and selected for the extremities of response (see [10]).

Patients harboring gene fusions	*N* (%)		*P* value^§^
	Long PFS (14)	Short PFS (26)	
Absence	4/14	12/26	0.3295^§^
Presence	10/14	14/26	
1 for each patient	5/10	10/14	0.4028^§^
>1 for each patient	5/10	4/14	
Only mRNA in the fusion	6/10	13/14	0.1222^§^
LncRNA in the fusion	8/10	2/14	0.0027^§^
CD274/PDCD1LG2 fusion	0/10	7/14	0.0188^§^

^§^The *P* values are reported as the two-sided Fisher exact test.
