# Supplementary material for: Are Fusion Transcripts in Relapsed/Metastatic Head and Neck Cancer Patients Predictive of Response to Anti-EGFR Therapies?
Source: Dis Markers. 2017 Nov 12;2017:6870614. doi: 10.1155/2017/6870614 (PMC5702394; doi:10.1155/2017/6870614)
Supplement: Supplementary file 1 — Supplementary Table 1. Details of the 27 different fusion transcripts that identified based on the applied workflow of analysis. [file 6870614.f1.pdf]

Supplementary Table 1:

gene-fusion	Category	Chromosome location of the original genes	type of fusion	annotation of left partner	annotations of right partner	Fusion transcripts containing one/both identified fusion partners §	
						other cancers	HNSCC
METTL13/DNM3	mRNA-mRNA	1q24.3/1q24.3	intra-chromosomal	METTL13 encodes for methyltransferase like 13 implicated in mRNA biogenesis, decay, and translation control.	DNM3 encodes for dynamin, a member of a family of guanosine triphosphate (GTP)-binding proteins that associate with microtubules and are involved in vesicular transport.	METTL13/EIF4G3 C1orf-9/DNM3	no
RCSD1/MPZL1	mRNA-mRNA	1q24.2/1q24.2	intra-chromosomal	RCSD1 encodes for RCSD domain containing 1 protein.	MPZL1, also known as PZL, encodes for myelin protein zero like 1, an immunoglobulin superfamily protein that specifically binds tyrosine phosphatase SHP-2 through its intracellular immunoreceptor tyrosine-based inhibitory motifs.	ATP1B1/RCSD1; MPZL1/PDE4DIP; DCAF6/MPZL1	no
CTNNA2/HES1	mRNA-mRNA	2p12-3/3q29	inter-chromosomal translocation	CTNNA2 encodes for catenin alfa cell-cell adhesion molecule that may function as a linker between cadherin adhesion receptors and the cytoskeleton to regulate cell-cell adhesion and differentiation in the nervous system.	HES1 encodes for hes family bHLH transcription factor 1. The protein is a transcriptional repressor of genes that require a bHLH protein for their transcription.	HIBCH/CTNNA2 PIGN/CTNNA2; MRLP35/CTNNA2 POLR1A/CTNNA2	GLIS3/CTNNA2
ZC3H15/ITGAV	mRNA-mRNA	2q32.1/2q32.1	intra-chromosomal	ZC3H15 encodes a potassium-dependent GTPase	ITGAV encodes the integrin subunit alfa V. The heterodimer consisting of alpha V and beta 3 subunits is also known as the vitronectin receptor. This integrin may regulate angiogenesis and cancer progression	ZC3H15/SERINC1 ZC3H15/CPS1; ZC3H15/GULP1; ITGAV/PDE11A; ITGAV/FARP2	no

FGF12/MB21D2	mRNA-mRNA	3q28-q29/3q29	intra-chromosomal	FGF12 encodes fibroblast growth factor 12. FGF family members possess broad mitogenic and cell survival activities. This growth factor lacks the N-terminal signal sequence present in most of the FGF family members, but it contains clusters of basic residues that have been demonstrated to act as a nuclear localization signal.	MB21D2 encodes for Mab-21 domain containing 2	FGF12/SYN3; TSC22D2/FGF12; C1orf196/FGF12	no
FLNB/ENSG00000245384	mRNA-lncRNA	3p14.3/4	inter-chromosomal translocation	FLNB encodes a member of the filamin family, filamin b that interacts with glycoprotein Ib alpha as part of the process to repair vascular injuries.	antisense lnc-TET2-4	FLNB/SLMAP; CCDC66/FLNB; ABHD6/FLNB	no
C9/RCOR1	mRNA-mRNA	5p13.1/14q32.31-q32.32	inter-chromosomal translocation	C9 encodes for the final component of the complement system	RCOR1 encodes a protein that is well-conserved, downregulated at birth, and with a specific role in determining neural cell differentiation. The encoded protein binds to the C-terminal domain of REST (repressor element-1 silencing transcription factor).	RICTOR/C9; LIFR/C9; FAM157A/C9	no
RPS6KA2/RNASET2	mRNA-mRNA	6q27/6q27	intra-chromosomal	RPS6KA2 encodes a member of the RSK (ribosomal S6 kinase) family of serine/threonine kinases. This kinase contains two non-identical kinase catalytic domains and phosphorylates various substrates, including members of the mitogen-activated kinase (MAPK) signalling pathway.	RNASET2 encodes a novel member of the Rh/T2/S-glycoprotein class of extracellular ribonucleases.	RPS6KA2/PSMG4	no

ANK1/KAT6A	mRNA-mRNA	8p11.21/8p11.21	intra-chromosomal	ANK1 encodes for ankirin 1, prototype of a family of proteins that link the integral membrane proteins to the underlying spectrin-actin cytoskeleton and play key roles in activities such as cell motility, activation, proliferation, contact and the maintenance of specialized membrane domains.	KAT6A encodes for lysine acetyltransferase 6A, a member of the MOZ, YBFR2, SAS2, TIP60 family of histone acetyltransferases that acts as a co-activator for several transcription factors	ANK1/FUT10; ANK1/ADAM7; NECAP1/ANK1; TMEM188/ANK1; WHSC1L1/ANK1; IKBKB/ANK1; KIAA0146/ANK1; PPFIA3/ANK1; KAT6A/IKBKB; KAT6A/KLK4; KAT6A/MSR1; KAT6A/ADAM2; KAT6A/GOT1L1; KAT6A/RAB11F1P1 FAM49B/KAT6A; CHD7/KAT6A; CREBBP/KAT6A	no
PVT1/ENSG00000253288	lncRNA-lncRNA	8q24.21/8	intra-chromosomal	This gene represents a long non-coding RNA locus that has been identified as a candidate oncogene.	intergenic LNC-FAM135B-1:1	no	no
CD274/PDCD1LG2	mRNA-mRNA	9p24.1/9p24.1	intra-chromosomal	CD274 encodes for programmed death ligand 1, PD-L1, an immune inhibitory receptor ligand that is expressed by hematopoietic and non-hematopoietic cells, such as T cells and B cells and various types of tumor cells.	PDCD1LG2 encodes for programmed cell death 1 ligand 2, PD-L2	KIAA1432/PDCD1LG2	no
MUSK/LPAR1	mRNA-mRNA	9q31.3/9q31.3	intra-chromosomal	MUSK encodes a muscle-specific tyrosine kinase receptor. The encoded protein may play a role in clustering of the acetylcholine receptor	LPAR1 encodes for a lysophosphatidic acid (LPA) receptor from a group known as EDG receptors that mediate diverse biologic functions, including proliferation, platelet aggregation, smooth muscle contraction, inhibition of neuroblastoma cell differentiation,	NAA15/LPAR1; SNX30/LPAR1	no

					chemotaxis, and tumor cell invasion.		
DLG2/PICALM	mRNA-mRNA	11q14.1/11q14.2	intra-chromosomal	DLG2 encodes for discs large MAGUK scaffold protein 2, a member of the membrane-associated guanylate kinase (MAGUK) family. The encoded protein forms a heterodimer with a related family member that may interact at postsynaptic sites to form a multimeric scaffold for the clustering of receptors, ion channels, and associated signaling proteins	PICALM encodes for phosphatidylinositol binding clathrin assembly protein that may be required to determine the amount of membrane to be recycled, possibly by regulating the size of the clathrin cage.	LRP5/DLG2; ARRB1/DLG2; INPPSA/DLG2: PLXNB2/DLG2: PICALM/RP11-849H4.2; PICALM/MLLT10; MLLT10/PICALM; ARRB1/PICALM	no
NUMA1/GRIA3	mRNA-mRNA	11q13.4/Xq25	inter-chromosomal translocation	NUMA1encodes for a large protein interacting with microtubules and playing a role in the formation and organization of the mitotic spindle during cell division.	GRIA3 encodes for glutamate ionotropic receptor AMPA type subunit 3. The subunit encoded by this gene belongs to a family of AMPA (alpha-amino-3-hydroxy-5-methyl-4-isoxazole propionate)-sensitive glutamate receptors, and is subject to RNA editing (AGA->GGA; R->G).	NUMA1/FAM83B; NUMA1/ADARB2; NUMA1/PTH; NUMA1/AATF; NUMA1/PIPTNM1; NUMA1/PPP2R5B; NUMA1/IQSEC3; NUMA1/TRPT1; NUMA1/ADHB12; NUMA1/CRYM; NUMA1/CCT5; SHANK2/NUMA1; STAG2/GRIA3	NUMA1/SFN
PPP6R3/MLL	mRNA-mRNA	11q13.2/11q23.3	intra-chromosomal	SAPS3 encodes for the phosphatase regulatory subunit 3 that modulates the activity of phosphatase-6 catalytic subunit by restricting substrate specificity, recruiting substrates, and determining the intracellular localization of the holoenzyme.	MLL,also known as KMT2A lysine methyltransferase, encodes a transcriptional coactivator that plays an essential role in regulating gene expression during early development and hematopoiesis.	PPP6R3/YAP1; PPP6R3/POLD3; PPP6R3/SSH2; PPP6R3/FADD; PPP6R3/SPTBN3; PPP6R3/MTL5; PPP6R3/LRP5; PPP6R3/ARHGAP1 RNF121/PPP6R3; KDM2A/PPP6R3; STRN3/PPP6R3; PC/PPP6R3; RAB11A/PPP6R3; CCDC132/PPP6R3;	no

						MLL/ATP5L; MLL/SLC22A10; MLL/CD86; MLL/MLLT10; MLL/ELL; MLL/MLLT4; MLL/MLLT3; MLL/IGHMBP2: MLL/FYN; MLL/ZDHHC7; MLLT4/MLL;	
ENSG00000231121/NAV3	lncRNA-mRNA	12/12q21.2	intra-chromosomal	lnc-NAV3	NAV3 belongs to the neuron navigator family and is expressed predominantly in the nervous system. The encoded protein contains coiled-coil domains and a conserved AAA domain characteristic for ATPases associated with a variety of cellular activities.	CPSF6/NAV3; PDK3/NAV3	no
ZMYM2/TRIM28	mRNA-mRNA	13q12.11/19q13.43	inter-chromosomal translocation	ZMYM2 encodes for zinc finger MYM-type containing 2, also named ZNF118 that may act as a transcription facto and be part of a BHC histone deacetylase complex.	TRIM28 encodes tripartite motif containing 28 protein that mediates transcriptional control by interaction with the Kruppel-associated box repression domain found in many transcription factors. The protein localizes to the nucleus and is thought to associate with specific chromatin regions.	ZMYM2/MTMR6; ZMYM2/KIAA0564; ZMYM2/DMRT2; TRIM28/ZBTB45	no
TRAF3/ENSG00000259717	mRNA-lncRNA	14q32.32/14	intra-chromosomal	TRAF3 encodes for TNF receptor associated factor 3, a member of the TNF receptor associated factor (TRAF) protein family. TRAF proteins associate with, and mediate the signal transduction from members of the TNF receptor (TNFR)	long intergenic non-protein coding RNA 677	TRAF3/MYO16; TRAF3/RCOR1; TRAF3/SFXN1; TRAF3/BMP3; TRAF3/WDR20; SLC22A23/TRAF3;	TRAF3/CDC42BPB

				superfamily. This protein participates in the signal transduction of CD40, a TNFR family member important for the activation of the immune response.			
ENSG00000259446/RYR3	lncRNA-mRNA	15/15q13.3-q14	intra-chromosomal	intergenic lncRNA (lnc-FMN1-4)	The protein encoded by this gene is a ryanodine receptor, which functions to release calcium from intracellular storage for use in many cellular processes.	RYR3/LARP4; RYR3/BUB1B; LARP4/RYR3	no
WDR90/RHOT2	mRNA-mRNA	16p13.3/16p13.3	intra-chromosomal	WDR90 encodes for WD repeat domain 90 whose function in humans in not well known. Given its broad expression in many tissues it could play a role in histone modifications, vesicular transport, or transcription regulation	This gene encodes a member of the Rho family of GTPase localized to the outer mitochondrial membrane and plays a role in mitochondrial trafficking and fusion-fission dynamics	WDR90/RAB40C; KIAA1731/WDR90 ; ROTH2/DPEP1	no
CLTC/RPS6KB1	mRNA-mRNA	17q23.1/17q23.1	intra-chromosomal	CLTC encodes for Clathrin heavy chain. Clatrin is the major protein component of the cytoplasmic face of intracellular organelles, called coated vesicles and coated pits. These specialized organelles are involved in the intracellular trafficking of receptors and endocytosis of a variety of macromolecules.	RPS6KB1 encodes a member of the ribosomal S6 kinase family of serine/threonine kinases. The protein responds to mTOR (mammalian target of rapamycin) signaling to promote protein synthesis, cell growth, and cell proliferation.	CLTC/FAM129C; CLTC/OTOP3; CLTC/CEP95; CLTC/USP32; CLTC/ROS1; CLTC/PTRH2; PTRH2/CLTC; USP32/CLTC; NF1/CLTC; VMP1/CLTC; RPS6KB1/VMP1; RPSK6KB1/TEX2; RPS6KB1/EFCAB3 RPS6KB1/RNF213; RPS6KB1/TUBD1; DDX42/RPS6KB1; C1orf132/RPS6KB1 BCAS3/RPS6KB1; TUBD1/RPS6KB1	RPS6KB1/VMP1

<table>
<tr><td>ZBTB7A/MAP2K2</td><td>mRNA-mRNA</td><td>19p13.3/19p13.3</td><td>intra-chromosomal</td><td>ZBTB7A encodes for zinc finger and BTB domain containing 7A, also known as Pokemon. The protein: plays a key role in the instruction of early lymphoid progenitors to develop into B lineage by repressing T-cell instructive Notch signals; specifically represses the transcription of the CDKN2A gene</td><td>MAP2K2 encode s for a dual specificity protein kinase that belongs to the MAP kinase kinase family. This kinase is known to play a critical role in mitogen growth factor signal transduction. It phosphorylates and thus activates MAPK1/ERK2 and MAPK2/ERK3.</td><td>ZBTB7A/TNK1; MAP2K2/CAMKK1</td><td>no</td></tr>
<tr><td>TPTE/BAGE2</td><td>mRNA-mRNA</td><td>21p11.2/21p11.2</td><td>intra-chromosomal</td><td>TPTE encodes for a transmembrane phosphatase with tensin homology, a PTEN-related tyrosine phosphatase which may play a role in the signal transduction pathways of the endocrine or spermatogenic function of the testis.</td><td>B melanoma antigen family member 2</td><td>no</td><td>no</td></tr>
<tr><td>IGLV1-40/IGLL5</td><td>mRNA-mRNA</td><td>22q11.22/22q11.21</td><td>Intra-chromosomal Inversion</td><td>IGLV1-40 encodes for immunoglobulin lambda variable 1-40</td><td rowspan="2">IGLL5 encodes one of the immunoglobulin lambda-like polypeptides. It is located within the immunoglobulin lambda locus but it does not require somatic rearrangement for expression. The first exon of this gene is unrelated to immunoglobulin variable genes; the second and third exons are the immunoglobulin lambda joining 1 and the immunoglobulin lambda constant 1 gene segments.</td><td>no</td><td>no</td></tr>
<tr><td>BMS1P20/IGLL5</td><td>mRNA-mRNA</td><td>22q11.21/22q11.21</td><td>intra-chromosomal</td><td>BMSP1P20 encodes for BMS1, ribosome biogenesis factor pseudogene 20. This locus appears to be a transcribed pseudogene of the BMS1 homolog of yeast ribosome assembly protein on chromosome 10 that has arisen from duplication of the 3' half of the parent gene. This gene lies in the immunoglobulin lambda gene cluster</td><td>no</td><td>no</td></tr>
</table>

PI4KA/CRKL	mRNA-mRNA	22q11.21/22q11.21	intra-chromosomal	This gene encodes a phosphatidylinositol (PI) 4-kinase which catalyzes the first committed step in the biosynthesis of phosphatidylinositol 4,5-bisphosphate. The protein encoded by this gene is a type III enzyme that is not inhibited by adenosine.	This gene encodes a protein kinase containing SH2 and SH3 (src homology) domains which has been shown to activate the RAS and JUN kinase signaling pathways and transform fibroblasts in a RAS-dependent fashion. It is a substrate of the BCR-ABL tyrosine kinase, plays a role in fibroblast transformation by BCR-ABL, and may be oncogenic	PI4KA/FAM19A5; PI4KA/SNAP29; PI4KA/ZNF274; PI4KA/RREB1; PI4KA/PRODH; PI4KA/FAM175A; PI4KA/DEPDC5; MED15/PI4KA; DGCR8/CRKL	no
ENSG00000231669/MSN	lncRNA-mRNA	X/Xq12	intra-chromosomal	intergenic lncRNA (lnc-MSN-1)	MSN encodes for moesin (membrane-organizing extension spike protein), a member of the ERM family which includes ezrin and radixin. ERM proteins appear to function as cross-linkers between plasma membranes and actin-based cytoskeletons. Moesin is localized to filopodia and other membranous protrusions that are important for cell-cell recognition and signaling and for cell movement.	MSN/MLRC4; MSN/HTR3D	no

§ according tothe TCGA database: http://54.84.12.177/PanCanFusV2/
